# Pediatric Systemic Lupus Erythematosus: Learning From Longer Follow Up to Adulthood

**DOI:** 10.3389/fped.2018.00144

**Published:** 2018-05-16

**Authors:** Giorgio Costagliola, Marta Mosca, Paola Migliorini, Rita Consolini

**Affiliations:** ^1^Laboratory of Immunology, Division of Pediatrics, Department of Clinical and Experimental Medicine, University of Pisa, Pisa, Italy; ^2^Rheumatology Unit, Department of Clinical and Experimental Medicine, University of Pisa, Pisa, Italy; ^3^Clinical Immunology and Allergy Unit, Department of Clinical and Experimental Medicine, University of Pisa, Pisa, Italy

**Keywords:** systemic lupus erythematosus, children, disease activity, organ damage, corticosteroids, thrombocytopenia, lupus nephritis, long-term follow-up

## Abstract

**Background:** Pediatric systemic lupus erythematosus (pSLE) is a rare condition, representing approximately 10% of SLE cases. The aim of this study was to identify variables to improve the diagnostic awareness and management of pSLE patients.

**Methods:** This retrospective study included 25 patients diagnosed with pSLE and followed at the University of Pisa. We collected data about clinical profile at disease onset and during a long-term follow-up, including disease activity, organ damage development, and treatments received.

**Results:** The mean patient age at disease onset was 14.6 ± 1.6 years, and the mean follow-up period was 14.17 ± 8.04 years. The most common initial manifestations were arthritis, malar rash, and cytopenias. The median time to diagnosis since the first symptoms was 6 months, and was significantly longer in patients with hematological onset (54 months). During follow-up, the number of patients with renal involvement showed a significant increase, from 36% at diagnosis to 72.2% after 10 years of disease evolution. Patients who developed chronic organ damage maintained a higher time-averaged disease activity during follow-up and received a significantly higher dose of corticosteroids.

**Conclusion:** Patients with immune cytopenia represent a group deserving strict clinical follow-up for the risk of evolution to SLE. Intense surveillance of renal function, early treatment and steroid-sparing strategies should be strongly considered in the management of pSLE patients.

## Introduction

Systemic lupus erythematosus (SLE) is an autoimmune disease with multisystemic involvement and a chronic-relapsing course.

According to the definition proposed by Silva et al, pediatric systemic lupus erythematosus (pSLE) identifies a subset of patients with disease onset of SLE prior to the 18th birthday (1), as this threshold reflects differences in clinical expression, disease activity and gender distribution.

The mean age at the onset of SLE symptoms is typically between 20 and 40 years, and therefore pSLE is a rare condition, representing approximately 10% of SLE cases. Pediatric-onset disease is also characterized by a less pronounced female/male ratio compared to the adult-onset disease (aSLE) (2–5).

Concerning the clinical phenotype, most studies have reported substantial differences between pSLE and adult-onset disease, showing a higher frequency of renal, hematological and neuropsychiatric involvement in pediatric patients, while chronic cutaneous lupus is more commonly diagnosed in patients with aSLE. The most relevant feature is the high rate of renal involvement, described in more than 65% of pSLE patients, while adult-onset disease is associated with a 33–55% rate of renal disease (6–10). Patients with pSLE also have higher disease activity compared to aSLE patients and frequently develop more organ damage, showing worse outcomes (11, 12).

However, while there are many studies concerning aSLE, studies on pSLE are often limited because of the small number of patients or the short duration of follow-up (13–16) in which the evaluation is complicated by the need for transition to adult care.

In the present study, we report the analysis of the clinical phenotype, disease activity and cumulative organ damage at disease onset and during long-term follow-up in a cohort of patients diagnosed with pSLE.

The aim of the present study is to identify variables that may improve the diagnostic sensibility and management of pSLE patients, by the analysis of the most common patterns observed at disease onset, the manifestations occurring during the clinical course, the factors associated with the development of organ damage and the effects of treatment received.

## Materials and methods

### Patient selection

The present study includes patients diagnosed with pSLE from 1980 to 2016 and followed in the Pediatrics, Rheumatology, and Clinical Immunology units of Santa Chiara Hospital at the University of Pisa.

Only patients who fulfilled 1997 ACR diagnostic criteria (17) were included in the present study. Patients with incomplete clinical documentation of the disease onset were excluded.

### Retrospective data collection

For each patient, we investigated initial disease manifestations, time to diagnosis (from first symptom to diagnosis) and the clinical expression of pSLE at the moment of diagnosis and during follow-up. Data on the follow-up were collected at 1, 3, 5, and 10 years after diagnosis and included the assessment of disease activity and organ damage and the treatment received.

### Definition and analysis of organ involvement

Acute cutaneous SLE was defined by the presence of malar rash, photosensitivity or both.

Articular involvement was defined by the presence of synovitis affecting two or more joints.

Hematologic involvement was defined by the presence of either 1 of the following: hemolytic anemia, thrombocytopenia (platelet count <100.000mm^3^), leukopenia or lymphopenia (leucocyte count <4,000/mm^3^ or lymphocyte count <1,500/mm^3^).

Renal involvement was defined by the presence of proteinuria >0.5 g in 24 h, hematuria or histologically documented renal damage. Renal biopsies were classified according to the International Society of Nephrology and the Renal Pathology Society classification (18).

Neuropsychiatric manifestations were classified according to American College of Rheumatology nomenclature of 1999 (19).

Data about specific organ involvement at diagnosis and after 10 years of follow-up were compared, in order to show the evolution of the clinical phenotype.

### Treatment received

The analysis focused on the use of immunosuppressive drugs and the mean annual dose of prednisone administered to each patient. Moreover, we investigated the infection occurrence and its correlation with the use of immunosuppressive drugs and, particularly, corticosteroids, dividing patients in two groups according to the presence/absence of infectious events and comparing the mean annual dose of prednisone between the two groups.

### Assessment of disease activity

Data on the disease activity at diagnosis and during follow-up were retrospectively calculated by using the revised version of the Systemic Lupus Erythematosus Disease Activity Index (SLEDAI-2K) (20). The validity of the retrospective assessment of disease activity was demonstrated in 1996 by Arce-Salinas et al and confirmed in 1999 by Arce-Salinas et al. (21) and FitzGerald et al. (22).

### Assessment of organ damage

Organ damage was evaluated and the Systemic Lupus International Collaborating Clinics/ American College of Rheumatology (SLICC/ACR) Damage Index was retrospectively calculated (23). We also investigated the role of the overall disease activity and of the use of corticosteroids in the development of organ damage: the population of patients with at least 10 years of follow-up was divided according to the presence of organ damage (“damage group” and “non-damage group”), and time-averaged SLEDAI scores and mean annual dose of prednisone were compared between the two groups of patients.

### Statistical analysis

The data on the categorical variables are reported as the percentage and absolute value. The data on the continuous variables with normal distribution (skewness between +1 and −1) are presented as the mean value and standard deviation. In case of asymmetric distribution, the data are presented as median value. An exact Fisher test was used to compare data about categorical variables from two groups. A non-parametric Mann-Whitney test or a T-Student test was used to compare the data about continuous from two groups of patients; a *p*-value of < 0.05 was considered significant.

## Results

### Demographic data

We analyzed the clinical reports of 32 patients with diagnosis of pSLE; seven of them were excluded for the absence of clinical documentation of the disease onset. The study included the remaining 25 patients (24 females and 1 male), diagnosed between 1980 and 2016 (4 from 1980 to 1995; 6 from 1996 to 2005; 15 from 2006 to 2016). The mean age at disease onset was 14.6 ± 1.6 years; 24 patients were of caucasian ethnicity and 1 patient was of hispanic ethnicity.

Data on the disease onset and clinical profile at diagnosis were available for the overall population. Follow-up data were available for 24 patients (one patient was excluded as very recently diagnosed), with a mean follow-up period of 14.17 ± 8.04 years. Follow-up data at 10 years from diagnosis were available for 18 patients, while for 13 patients the follow-up period was longer than 10 years.

### Clinical manifestation at disease onset

In the present cohort, arthritis was the most common initial manifestation, being observed in 12 out of 25 patients (48%), and malar rash was detected in 10 patients (40%). Hematological involvement was observed in 7 patients at disease onset (28%), and among these patients, autoimmune cytopenia was the only first sign of pSLE for 4 patients (3 thrombocytopenia patients and 1 hemolytic anemia patient).

Renal involvement was observed in 5 patients (20%). Other manifestations observed include Raynaud's phenomenon (2 patients), xerostomia and xerophthalmia (1 patient), pleuro-pericarditis (1 patient), gastro-enteritis (1 patient), and deep venous thrombosis (1 patient).

### Time to diagnosis

The median time to diagnosis was 6 months.

The 4 patients with only cytopenia at disease onset had a significantly longer median time to diagnosis (54 months) compared to patients with other clinical manifestations of pSLE (*p* = 0.02), and serum ANA and anti-dsDNA positivity appeared later than hematological involvement. The median time between the onset of cytopenia and the development of other symptoms in this subgroup of patients was 27 months.

Patients who initially presented with non-SLE specific signs (Raynaud's phenomenon, xerostomia and xerophthalmia, and gastroenteritis) had a mean time to diagnosis of 21.5 months (*p* = 0.03).

### Clinical profile at diagnosis

At diagnosis, the most common manifestations observed were arthritis (16 patients, 64% of our cohort), hematological involvement (15 patients, 60%), and acute cutaneous lupus (16 patients, 64%): malar rash and photosensitivity were identified, respectively, in 13 patients (52%), and 10 patients (40%).

Among hematological manifestations, leukopenia was observed in 10 patients (40%), thrombocytopenia was observed in 7 patients (28%), and hemolytic anemia was observed in 6 patients (24%).

Renal involvement was detected in 9 patients (36%), while mucosal ulcerations were detected in 12%, and alopecia was detected in 16% of patients.

Moreover, serositis was observed in 2 patients: one patient developed pericarditis, and the other patient developed pleuro-pericarditis.

In the present cohort, we did not identify any cases of neuropsychiatric involvement or manifestations of chronic cutaneous lupus, such as discoid rash, at diagnosis.

### Clinical profile during follow-up

During follow-up, we registered a significant decrease in the percentage of patients with cutaneous and articular involvement, and in the present cohort we did not observe any cases of erosive arthritis.

We also documented a statistically significant increase of renal involvement form diagnosis to 10-year follow-up (from 36 to 72%). Renal biopsy was performed in 11 patients (44% of our cohort): histological evaluation showed 1 case of class II glomerulonephritis, 2 cases of class III glomerulonephritis and 8 cases of class IV glomerulonephritis. Patients who received biopsy had a mean age at disease onset of 13.9 ± 1.8 years, while patients who did not receive biopsy had a mean age at disease onset of 15.2 ± 1.2 years (*p* = 0.02). In the subgroup of patients with histologically documented lupus nephritis, 3 out of 11 patients (26.7%) developed end stage renal disease (ESRD), and one of these patients required dialysis.

Neuropsychiatric lupus (NPSLE), including episodes of lupus headache, seizures and two cases of cranial neuropathy (one case involving the optical nerve, and the other case involving the oculomotor nerve) due to central nervous system vasculitis, was less frequently observed. After 1 year of follow-up, one patient received a diagnosis of “multi-infarct encephalopathy” based on an evaluation with brain magnetic resonance.

**Figure 1:**
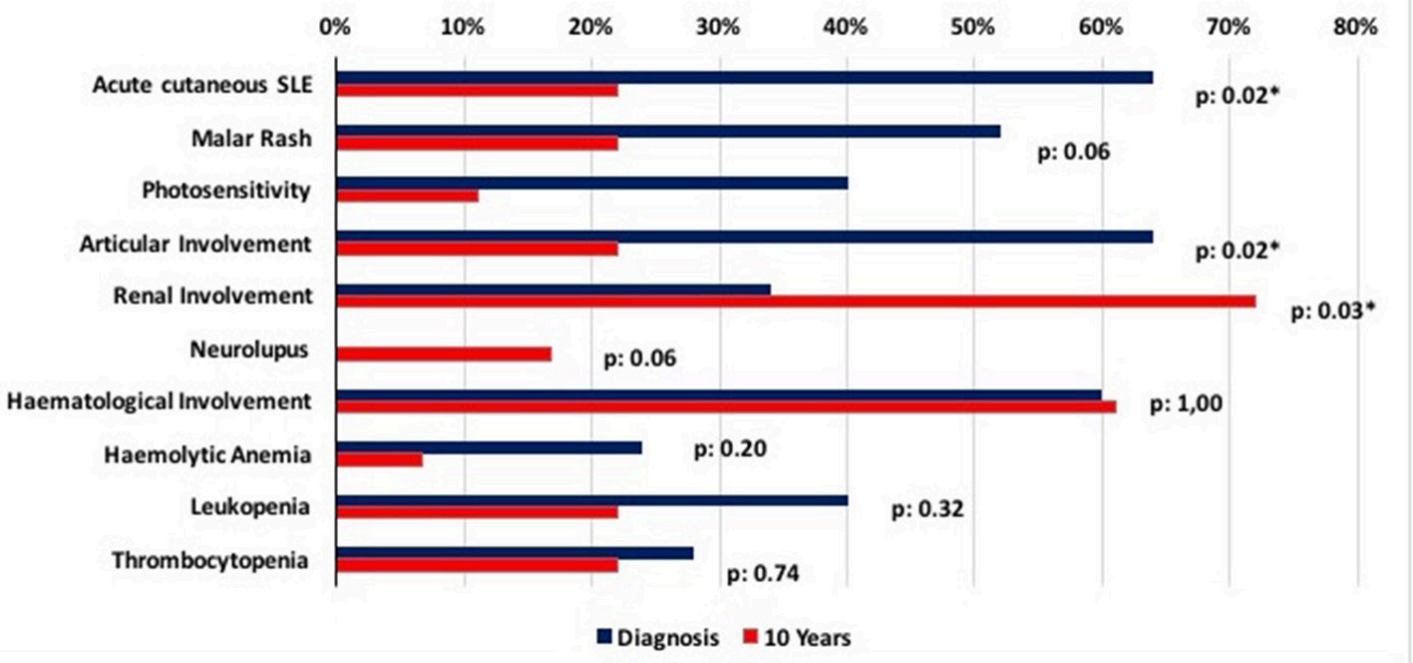
Differences in clinical profile at diagnosis and after 10 years of follow-up. Clinical data collected at diagnosis and after 10 years of follow-up are compared, highlighting the statistically significant reduction of articular and cutaneous involvement and the growth in the percentage of patients with renal involvement.

Table 1 summarizes the clinical manifestations at diagnosis and during the follow-up for the present cohort at 1, 3, 5, and 10 years from the diagnosis of pSLE, and Figure 1 highlights the comparison between clinical profile at diagnosis and after 10 years of follow-up.

**Table 1:** Evolution of clinical profile during follow-up.

**Clinical feature**	**Diagnosis**	**1 Year**	**3 Years**	**5 Years**	**10 Years**
Acute cutaneous SLE	16/25 (64%)	9/24 (37.5%)	7/24 (29%)	6/20 (30%)	4/18 (22%)
Malar rash	13/25 (52%)	6/24 (25%)	6/24 (25%)	5/20 (25%)	4/18 (22%)
Photosensitivity	10/25 (40%)	8/24 (33%)	5/24 (21%)	5/20 (25%)	2/18 (11%)
Articular involvement	16/25 (64%)	6/24 (25%)	6/24 (25%)	4/20 (20%)	4/18 (22%)
Renal involvement	9/25 (34%)	12/24 (50%)	11/24 (46%)	12/20 (60%)	13/18 (72%)
Neurolupus	0/25 (0%)	2/24 (8%)	1/24 (4%)	2/20 (10%)	3/18 (16,7%)
Hematological involvement	15/25 (60%)	11/24 (46%)	8/24 (33%)	8/20 (40%)	11/18 (61%)
Haemolytic anemia	6/25 (24%)	2/24 (8%)	1/24 (4%)	1/20 (5%)	1/18 (6.7%)
Leukopenia	10/25 (40%)	5/24 (21%)	5/24 (21%)	4/20 (20%)	4/18 (22%)
Thrombocytopenia	7/25 (28%)	4/24 (17%)	3/24 (13%)	3/20 (15%)	6/18 (33%)

*The analysis of the clinical manifestations at diagnosis and during follow-up at 1, 3, 5, and 10 years from diagnosis evidences the evolution of the clinical phenotype during the disease progression*.

### Therapy and infection occurrence

Twenty-three patients (92%) received corticosteroids and 20 patients (80%) received antimalarial drugs during the clinical course. The median annual dose of corticosteroids was 1.825 g/year of prednisone

Twenty patients (80%) received immunosuppressive drugs during follow-up. The most commonly administered drugs included azathioprine (48%), mycophenolate (40%), cyclophosphamide (36%), methotrexate (24%), cyclosporine (16%), and rituximab (16%).

During follow-up, 5 patients (20.8%) experienced clinically relevant infections, and 2 of these patients had multiple infections. Overall, we observed 3 cases of Cytomegalovirus (CMV) reactivation, 2 cases of Herpes-Zoster Virus reactivation, 1 primary severe infection by Epstein-Barr virus and 1 infection by Mycobacterium Tuberculosis.

The median annual dose of steroids for this subgroup of patients was 4.015 g/year, which was significantly higher compared to that for patients who did not develop infections (*p* = 0.03).

### Disease activity

Data about median disease activity at diagnosis and during follow-up are summarized in Table 2.

**Table 2:** Disease activity at diagnosis and during follow-up.

	**Diagnosis**	**1 Year**	**3 Years**	**5 Years**	**10 Years**
Median SLEDAI score	8	4	4	5	6

*The median SLEDAI (Systemic Lupus Erythematosus Disease Activity Index) score of our cohort of patients was high at diagnosis, and maintained a median value ≥4 points during the entire follow-up period*.

### Organ damage development

During the entire observation period, 15 patients (62.5%) developed non-reversible organ damage. Nine out of 24 patients (37.5%) developed renal damage, including nephrotic syndrome or ESRD, and 7 patients (29%) showed vascular involvement, with deep venous thrombosis or ischemic arterial lesions. The nervous system was involved in 4 patients (16.7%), with ischemic stroke, cranial neuropathies and cerebral vasculitis. Cutaneous damage, consistently observed as chronic non-scarring alopecia, was detected in 3 patients (15%), with muscular (myositis), cardiac (acute myocardial infarction), and pulmonary (restrictive syndrome and pulmonary heart) damages respectively identified in each patient.

We observed a progressive increase in the percentage of patients with organ damage and in the mean SLICC score of the population with damage. Table 3 summarizes the data on organ damage development and the mean SLICC score.

**Table 3:** Analysis of organ damage during follow-up.

	**1 Year**	**3 Years**	**5 Years**	**10 Years**	**>10 Years**
Patients with damage	5/24 (20.8%)	6/23 (26%)	10/20 (50%)	13/18 (72.2%)	11/13 (84.6%)
Median SLICC score	1	1	1	2	3

*The percentage of patients showing organ damage and the median SLICC (Systemic Lupus International Collaborating Clinics/American College of Rheumatology) score of our cohort of patients showed a progressive increase during the long-term follow-up*.

### Relationship between time-averaged disease activity and organ damage development

The median time-averaged disease activity of the patients of the “damage group” was 8 points, while time-averaged disease activity of patients of the “non-damage group” was 4 points (*p* = 0.04).

### Relationship between the mean dose of corticosteroids received and organ damage

Patients in the “damage group” required a median annual dose of steroids 3.5 g/year, significantly higher compared to those in the “non-damage group,” that was 1.14 g/year (*p* = 0.01).

## Discussion

The follow-up of pSLE patients has been scarcely investigated, as the delicate process of transition to adult rheumatology clinics makes it difficult to perform long-term studies.

In our hospital, the coexistence of the Pediatrics, Rheumatology, and Clinical Immunology units facilitated the follow up of the present patients for several years after clinical transition. The aim of the present study was to analyze the evolution of pSLE to identify key issues for an early intervention that could modify and improve long-term prognosis.

Analysis of disease onset in the overall population showed a clear predominance of articular and cutaneous manifestations as first signs of pSLE, and these two phenotypes were associated with an early diagnosis of lupus. Specifically, photosensitive malar rash is universally recognized as highly suggestive for lupus, and its presence consistently accelerates the formulation of diagnosis.

Articular involvement in pSLE is extremely common, but the clinical manifestation of arthritis do not evoke a specific pattern in SLE; therefore, when arthritis is the first sign of pSLE, making a correct diagnosis may pose some difficulties. However, we suggest that, even at pediatric age, SLE must be included in the potential diagnosis in cases of unexplained arthritis, particularly when associated with abnormal hematologic values.

Renal involvement at presentation was also associated with a short time to pSLE diagnosis, partially depending on an early recourse to renal biopsy.

Hematological manifestations were common at disease onset, but when immune-mediated cytopenia was the only initial sign of pSLE, the diagnostic time was significantly longer. These manifestations, particularly autoimmune thrombocytopenia in adolescents, may eventually manifest as SLE many years after clinical onset. Therefore, patients with immune-mediated cytopenia deserve a strict clinical follow-up, with periodical determination of anti-nuclear antibodies (ANA), anti-dsDNA autoantibodies, and anti-phospholipid antibodies.

Compared with data from previous studies on adult-onset disease, the present pSLE cohort had a greater percentage of patients with malar rash, photosensitivity and renal involvement, while no differences were observed relative to the incidence of articular and hematological manifestations (24–26). In the present cohort, while articular, hematologic, cutaneous and renal involvements were frequent at diagnosis, no patients showed neuropsychiatric signs or symptoms. This finding is in disagreement with the current literature, which reports significant central nervous system involvement in patients with pSLE. However, the retrospective method of the present study makes it difficult to ascribe a neurological manifestation to pSLE, and it is likely that we underestimated the actual prevalence of neurological involvement in the present cohort.

As observed in the present cohort, non-lupus specific onset is more common in pSLE than in adult-onset disease: these patients commonly show a long time to diagnosis, particularly dependent on a progressive disease evolution. Previous studies have reported other various non-specific patterns of pSLE onset, including recurring abdominal pain, acute abdomen, coronary arteritis, chorea, cough, and parotitis (27–31). These data confirm the assumption that pSLE is a heterogeneous condition; therefore, according to Bader-Meunier et al. we suggest that in pediatric age, especially in adolescents, the diagnosis of SLE should be firmly considered in the presence of “not explained organ involvement and laboratory findings suggestive for systemic inflammation” (27).

Analysis of the follow-up of the present cohort revealed a significant increase in the percentage of patients with renal involvement: many studies confirm a higher incidence of renal damage in pSLE compared to aSLE (32), indicating that a strict follow-up of renal function in patients with pSLE is necessary. Moreover, is important to highlight that almost 20% of patients with histologically documented renal damage develop ESRD (11, 33). Therefore, evaluation of the mean age at disease onset of patients who received biopsy suggests that a younger age at disease onset could be related to a greater severity of renal involvement.

Evaluation of disease activity confirmed that pSLE is a condition characterized by high activity at diagnosis, with a higher mean SLEDAI score when compared with adult-onset disease. These findings are consistent with those of many previous studies, which report even higher scores (12, 34).

During follow up, as expected, we observed a considerable growth in the percentage of patients with organ damage and in the mean SLICC score. The observed damage was mainly renal, vascular and neurological, while muscular damage was detected in only one patient, and none of the patients showed skeletal damage.

The relationship between disease activity and organ damage showed that, independent from the mean SLEDAI score at disease onset, patients with chronically active disease were at significantly higher risk of developing organ damage; studies by Brunner and Sato showed similar conclusions, confirming that cumulative disease activity is a predictor for organ damage (35, 36). To our knowledge, the present study is the longest follow-up study investigating the relationship between disease activity and organ damage in pediatric-onset disease. The findings underline the necessity for early intervention in patients with pSLE to promptly reduce disease activity and prevent cumulative non-reversible organ damage.

In addition to the damage induced by the pathology, damage caused by the use of corticosteroids and immunosuppressive drugs must be considered. The clinical severity of organ involvement in pSLE and the high disease activity often lead to the use of immunosuppressive drugs, reported to be higher than in adult-onset disease (11). Interestingly, we identified a correlation between the administration of corticosteroids and the development of organ damage, indicating that corticosteroids are the most relevant contributors to organ damage among the immunosuppressive agents, confirming the data from several studies performed in adult and pediatric populations (37–39). Further studies on larger cohorts are needed to investigate the role of other immunosuppressive drugs in the development of organ damage, in order to optimize the treatment strategy for pSLE patients.

Early organ involvement, high disease activity and the considerable need for corticosteroids and immunosuppressive drugs make pSLE a challenge for clinicians, who should be trained on managing the complexity of a systemic disease with specific age-related differences and complications.

Despite limitations by the small number of patients and the retrospective method of analysis, the present study represents an effort to increase the current knowledge of the clinical picture, disease activity and organ damage development at disease onset and during follow-up of pSLE patients, and offers a great contribution for the early disease diagnosis and the prevention of severe complications. The new era of “treat to target” will enable the use of steroid-sparing strategies, opening a window of opportunity for improving outcomes in pediatric aged patients.

## Conclusion

The analysis of disease onset underlines that patients with autoimmune cytopenias represent a population deserving a closer surveillance for the possibility of evolving to lupus.

During follow-up we observed a marked increase in the percentage of patients with renal damage, and therefore an intense surveillance of renal function must be performed for pSLE patients, particularly those with early disease onset.

Our study evidenced a positive correlation between the development of organ damage and both time-averaged disease activity and use of corticosteroids, suggesting that early intervention to reduce disease activity could be useful in the prevention of chronic-non-reversible complications, and that steroid-sparing strategies should be accurately considered.

## Data availability

Data about clinical profile of the single patients are available in the additional file (Appendix 1). The other data are available on request contacting the corresponding author.

## Author contributions

All authors participated in the collection and interpretation of clinical data. GC wrote the paper. All authors contributed to manuscript revision, read and approved the submitted version.

### Conflict of interest statement

The authors declare that the research was conducted in the absence of any commercial or financial relationships that could be construed as a potential conflict of interest.
