# Supplementary material for: Pediatric Systemic Lupus Erythematosus: Learning From Longer Follow Up to Adulthood
Source: Front Pediatr. 2018 May 16;6:144. doi: 10.3389/fped.2018.00144 (PMC5964827; doi:10.3389/fped.2018.00144)
Supplement: Supplementary file 1 [file Data_Sheet_1.PDF]

Appendix 1

In this file are contained clinical data of the single patients of our cohort, in order to help to understand the peculiarities of the disease onset and the progressive evolution of the clinical phenotype during a long-term follow-up. Please note that the difference between clinical profile at presentation and at diagnosis derives from the long time between the disease onset and the diagnosis of pSLE.

Patient 1
Age at onset: 15 years
Clinical presentation: Gastroenteritis, malar rash, photosensitivity
Time to diagnosis: 17 months
Duration of follow-up: 4 years

Clinical feature	Diagnosis	1 Year	3 Years
Acute cutaneous SLE	X	X	X
Malar Rash			
Photosensitivity	X	X	X
Articular Involvement	X	X	X
Renal Involvement			
Neurolupus			
Haematological Involvement	X	X	X
Haemolytic Anemia	X		
Leukopenia	X	X	X
Thrombocytopenia			

Patient 2
Age at onset: 15 years
Clinical presentation: Photosensitivity, malar rash
Time to diagnosis:11 months
Duration of follow-up: 30 years

Clinical feature	Diagnosis	1 Year	3 Years	5 Years	10 Years
**Acute cutaneous SLE**	X				
**Malar Rash**	X				
**Photosensitivity**					
**Articular Involvement**	X	X			X
**Renal Involvement**		X	X	X	X
**Neurolupus**					
**Haematological Involvement**	X	X		X	X
**Haemolytic Anemia**					
**Leukopenia**	X	X		X	X
**Thrombocytopenia**					
**Other**					Pleuritis

Patient 3
Age at onset: 16 years
Clinical presentation: Arthritis
Time to diagnosis: 2 months
Duration of follow-up: 4 years

Clinical feature	Diagnosis	1 Year	3 Years
**Acute cutaneous SLE**	X	X	
**Malar Rash**	X	X	
**Photosensitivity**	X	X	
**Articular Involvement**	X	X	X
**Renal Involvement**		X	
**Neurolupus**			
**Haematological Involvement**			
**Haemolytic Anemia**			
**Leukopenia**			
**Thrombocytopenia**			

Patient 4
Age at onset: 17
Clinical presentation: Pleuritis, Pericarditis
Time to diagnosis: 1 month
Duration of follow-up: 7 years

Clinical feature	Diagnosis	1 Year	3 Years	5 Years
**Acute cutaneous SLE**				
**Malar Rash**				
**Photosensitivity**				
**Articular Involvement**				X
**Renal Involvement**	X	X	X	X
**Neurolupus**				
**Haematological Involvement**	X	X	X	
**Haemolytic Anemia**				
**Leukopenia**	X	X	X	
**Thrombocytopenia**				
**Other**	Pleuro-pericarditis		Pleuro-pericarditis	Pleuritis

Patient 5
Age at onset: 15 years
Clinical presentation: Thrombocytopenia
Time to diagnosis: 62 months
Duration of follow-up: 4 years

Clinical feature	Diagnosis	1 Year	3 Years
Acute cutaneous SLE	X	X	X
Malar Rash	X		X
Photosensitivity	X	X	X
Articular Involvement			
Renal Involvement			
Neurolupus			
Haematological Involvement	X	X	X
Haemolytic Anemia			
Leukopenia			
Thrombocytopenia	X	X	X

Patient 6
Age at onset: 14
Clinical presentation: Nephritis, malar rash, photosensitivity
Time to diagnosis: 0 months
Duration of follow-up: 24 years

Clinical feature	Diagnosis	1 Year	3 Years	5 Years	10 Years
**Acute cutaneous SLE**	X	X	X	X	
**Malar Rash**	X	X	X	X	
**Photosensitivity**	X	X		X	
**Articular Involvement**					
**Renal Involvement**	X	X	X	X	X
**Neurolupus**					X
**Haematological Involvement**					
**Haemolytic Anemia**					
**Leukopenia**					
**Thrombocytopenia**					
**Other**			Vasculitis		Vasculitis

Patient 7
Age at onset: 15 years
Clinical presentation: Arthritis, proteinuria, malar rash, cytopenia
Time to diagnosis: 0 months
Duration of follow-up: 24 years

Clinical feature	Diagnosis	1 Year	3 Years	5 Years	10 Years
Acute cutaneous SLE	X				
Malar Rash	X				
Photosensitivity					
Articular Involvement	X				
Renal Involvement	X	X	X	X	X
Neurolupus					X
Haematological Involvement	X	X	X	X	X
Haemolytic Anemia					
Leukopenia	X				
Thrombocytopenia	X	X	X	X	X

Patient 8
Age at onset: 14 years
Clinical presentation: Malar rahs, arthritis
Time to diagnosis: 0 months
Duration of follow-up: 17 years

Clinical feature	Diagnosis	1 Year	3 Years	5 Years	10 Years
**Acute cutaneous SLE**	X				
**Malar Rash**	X				
**Photosensitivity**					
**Articular Involvement**	X				
**Renal Involvement**				X	X
**Neurolupus**					
**Haematological Involvement**					
**Haemolytic Anemia**					
**Leukopenia**					
**Thrombocytopenia**					
**Other**			Pleuro-pericarditis		

Patient 9
Age at onset: 13 years
Clinical presentation: Malar rash
Time to diagnosis: 12 months
Duration of follow-up: 10 years

Clinical feature	Diagnosis	1 Year	3 Years	5 Years	10 Years
**Acute cutaneous SLE**	X	X	X	X	X
**Malar Rash**	X	X	X	X	X
**Photosensitivity**	X	X		X	X
**Articular Involvement**		X		X	X
**Renal Involvement**			X	X	X
**Neurolupus**					
**Haematological Involvement**				X	X
**Haemolytic Anemia**					
**Leukopenia**				X	X
**Thrombocytopenia**					

Patient 10
Age at onset: 16 years
Clinical presentation: Arthritis, Raynaud's phenomenon
Time to diagnosis: 26 months
Duration of follow-up: 19 years

Clinical feature	Diagnosis	1 Year	3 Years	5 Years	10 Years
Acute cutaneous SLE					
Malar Rash					
Photosensitivity					
Articular Involvement	X	X	X	X	
Renal Involvement					X
Neurolupus					X
Haematological Involvement					
Haemolytic Anemia					
Leukopenia					
Thrombocytopenia					

Patient 11
Age at onset: 12 years
Clinical presentation: malar rash
Time to diagnosis: 6 months
Duration of follow-up:not available (too recently diagnosed)

Clinical feature	Diagnosis
**Acute cutaneous SLE**	X
**Malar Rash**	X
**Photosensitivity**	X
**Articular Involvement**	X
**Renal Involvement**	
**Neurolupus**	
**Haematological Involvement**	
**Haemolytic Anemia**	
**Leukopenia**	
**Thrombocytopenia**	

Patient 12
Age at onset: 17 years
Clinical presentation: Arthritis, photosensitivity
Time to diagnosis: 0 months
Duration of follow-up: 5 years

Clinical feature	Diagnosis	1 Year	3 Years	5 Years
**Acute cutaneous SLE**	X	X	X	X
**Malar Rash**				
**Photosensitivity**	X	X	X	X
**Articular Involvement**	X		X	X
**Renal Involvement**				
**Neurolupus**				
**Haematological Involvement**	X	X		
**Haemolytic Anemia**				
**Leukopenia**	X	X		
**Thrombocytopenia**				

Patient 13
Age at onset: 15 years
Clinical presentation: Renal involvement
Time to diagnosis: 2 months
Duration of follow-up: 11 years

Clinical feature	Diagnosis	1 Year	3 Years	5 Years	10 Years
Acute cutaneous SLE					
Malar Rash					
Photosensitivity					
Articular Involvement					
Renal Involvement	X	X	X	X	X
Neurolupus					
Haematological Involvement				X	X
Haemolytic Anemia					
Leukopenia					
Thrombocytopenia				X	X

Patient 14
Age at onset: 14 years
Clinical presentation: Arthritis, Raynaud's phenomenon
Time to diagnosis: 12 months
Duration of follow-up: 15 years

Clinical feature	Diagnosis	1 Year	3 Years	5 Years	10 Years
**Acute cutaneous SLE**	X				
**Malar Rash**					
**Photosensitivity**	X				
**Articular Involvement**	X				
**Renal Involvement**					
**Neurolupus**					
**Haematological Involvement**	X	X	X	X	X
**Haemolytic Anemia**					
**Leukopenia**	X	X	X	X	X
**Thromobocytopenia**					
**Other**					Vasculitis

Patient 15
Age at onset: 17 years
Clinical presentation: Artritis, cytopenia
Time to diagnosis: o months
Duration of follow-up: 16 years

Clinical feature	Diagnosis	1 Year	3 Years	5 Years	10 Years
**Acute cutaneous SLE**					
**Malar Rash**					
**Photosensitivity**					
**Articular Involvement**	X				X
**Renal Involvement**		X	X	X	X
**Neurolupus**					
**Haematological Involvement**	X	X	X	X	X
**Haemolytic Anemia**	X	X			
**Leukopenia**	X		X	X	
**Thrombocytopenia**					X

Patient 16
Age at onset: 15 years
Clinical presentation: Thrombocytopenia
Time to diagnosis: 67 months
Duration of follow-up: 10 years

Clinical feature	Diagnosis	1 Year	3 Years	5 Years	10 Years
**Acute cutaneous SLE**					
**Malar Rash**					
**Photosensitivity**					
**Articular Involvement**			X		
**Renal Involvement**	X				X
**Neurolupus**					
**Haematological Involvement**	X	X	X	X	X
**Haemolytic Anemia**					
**Leukopenia**	X		X		
**Thrombocytopenia**	X	X	X	X	X
**Other**				Vasculitis	Vasculitis

Patient 17
Age at onset: 15 years
Clinical presentation: Arthritis, renal involvement
Time to diagnosis: 34 months
Duration of follow-up: 13 years

Clinical feature	Diagnosis	1 Year	3 Years	5 Years	10 Years
**Acute cutaneous SLE**			X		X
**Malar Rash**			X		X
**Photosensitivity**			X		X
**Articular Involvement**	X		X		
**Renal Involvement**	X	X	X	X	X
**Neurolupus**					
**Haematological Involvement**		X	X	X	X
**Haemolytic Anemia**		X	X	X	X
**Leukopenia**					
**Thrombocytopenia**					

Patient 18
Age at onset: 13 years
Clinical presentation: Thrombocytopenia
Time to diagnosis: 52 months
Duration of follow-up: 15 years

Clinical feature	Diagnosis	1 Year	3 Years	5 Years	10 Years
**Acute cutaneous SLE**	X				
**Malar Rash**	X				
**Photosensitivity**					
**Articular Involvement**	X				
**Renal Involvement**	X	X	X	X	X
**Neurolupus**					
**Haematological Involvement**	X				
**Haemolytic Anemia**					
**Leukopenia**					
**Thrombocytopenia**	X				

Patient 19
Age at onset: 16 years
Clinical presentation: Arthritis
Time to diagnosis: 10 months
Duration of follow-up: 10 years

Clinical feature	Diagnosis	1 Year	3 Years	5 Years	10 Years
**Acute cutaneous SLE**	X	X			
**Malar Rash**	X	X			
**Photosensitivity**					
**Articular Involvement**	X				
**Renal Involvement**		X	X	X	X
**Neurolupus**					
**Haematological Involvement**	X				
**Haemolytic Anemia**	X				
**Leukopenia**	X				
**Thrombocytopenia**	X				

Patient 20
Age at onset: 15 years
Clinical presentation: Malar rash
Time to diagnosis: 0 months
Duration of follow-up: 26 years

Clinical feature	Diagnosis	1 Year	3 Years	5 Years	10 Years
**Acute cutaneous SLE**	X	X	X	X	X
**Malar Rash**	X	X	X	X	X
**Photosensitivity**	X	X	X	X	
**Articular Involvement**					
**Renal Involvement**					
**Neurolupus**					
**Haematological Involvement**					
**Haemolytic Anemia**					
**Leukopenia**					
**Thrombocytopenia**					

Patient 21
Age at onset: 15 years
Clinical presentation: Malar rash, xerostomia, xerophtalmia
Time to diagnosis: 86 months
Duration of follow-up: 10 years

Clinical feature	Diagnosis	1 Year	3 Years	5 Years	10 Years
Acute cutaneous SLE	X			X	X
Malar Rash	X			X	X
Photosensitivity	X			X	
Articular Involvement					
Renal Involvement					
Neurolupus					
Haematological Involvement	X				X
Haemolytic Anemia					
Leukopenia					
Thrombocytopenia	X				X

Patient 22
Age at onset: 14 years
Clinical presentation: Arthritis, cytopenia
Time to diagnosis: 0 months
Duration of follow-up: 18 years

Clinical feature	Diagnosis	1 Year	3 Years	5 Years	10 Years
**Acute cutaneous SLE**					
**Malar Rash**					
**Photosensitivity**					
**Articular Involvement**	X				
**Renal Involvement**		X		X	X
**Neurolupus**		X	X	X	
**Haematological Involvement**	X				X
**Haemolytic Anemia**	X				
**Leukopenia**	X				
**Thrombocytopenia**					X

Patient 23
Age at onset: 11 years
Clinical presentation: Haemolytic anemia
Time to diagnosis: 35 months
Duration of follow-up: 3 years

Clinical feature	Diagnosis	1 Year	3 Years
Acute cutaneous SLE			
Malar Rash			
Photosensitivity			
Articular Involvement			
Renal Involvement	X	X	X
Neurolupus			
Haematological Involvement	X		
Haemolytic Anemia	X		
Leukopenia			
Thrombocytopenia			

Patient 24
Age at onset: 15 years
Clinical presentation: Deep venous thrombosis, arthritis
Time to diagnosis: 0 months
Duration of follow-up: 28 years

Clinical feature	Diagnosis	1 Year	3 Years	5 Years	10 Years
**Acute cutaneous SLE**		X		X	
**Malar Rash**				X	
**Photosensitivity**		X		X	
**Articular Involvement**	X	X			
**Renal Involvement**					
**Neurolupus**		X		X	X
**Haematological Involvement**	X	X			
**Haemolytic Anemia**	X				
**Leukopenia**					
**Thrombocytopenia**	X	X			

Patient 25
Age at onset: 11,5 years
Clinical presentation: Malar rash, arthritis, renal involvement
Time to diagnosis: 1 month
Duration of follow-up: 17 years

Clinical feature	Diagnosis	1 Year	3 Years	5 Years	10 Years
Acute cutaneous SLE	X				
Malar Rash	X				
Photosensitivity					
Articular Involvement	X				X
Renal Involvement	X				
Neurolupus					
Haematological Involvement					X
Haemolytic Anemia					
Leukopenia					X
Thrombocytopenia					
Other	Pericarditis				
